# Guidelines for public database submission of uncultivated virus genome sequences for taxonomic classification

**DOI:** 10.1038/s41587-023-01844-2

**Published:** 2023-07-01

**Authors:** Evelien M. Adriaenssens, Simon Roux, J. Rodney Brister, Ilene Karsch-Mizrachi, Jens H. Kuhn, Arvind Varsani, Tong Yigang, Alejandro Reyes, Cédric Lood, Elliot J. Lefkowitz, Matthew B. Sullivan, Robert A. Edwards, Peter Simmonds, Luisa Rubino, Sead Sabanadzovic, Mart Krupovic, Bas E. Dutilh

**Affiliations:** 1 Quadram Institute Bioscience, Norwich Research Park, Rosalind Franklin Road, Norwich, UK; 2 United States Department of Energy Joint Genome Institute, Lawrence Berkeley National Laboratory, Berkeley, CA, USA; 3 National Center for Biotechnology Information, National Library of Medicine, National Institutes of Health, Bethesda, MD, USA; 4 Integrated Research Facility at Fort Detrick, National Institute of Allergy and Infectious Diseases, National Institutes of Health, Fort Detrick, Frederick, MD, USA; 5 The Biodesign Center for Fundamental and Applied Microbiomics, Arizona State University, Tempe, AZ, USA; 6 Structural Biology Research Unit, Department of Clinical Laboratory Sciences, University of Cape Town, Cape Town, South Africa; 7 Beijing Advanced Innovation Center for Soft Matter Science and Engineering, College of Life Science and Technology, Beijing University of Chemical Technology, Beijing, China; 8 Max Planck Tandem Group in Computational Biology, Department of Biological Sciences, Universidad de los Andes, Bogotá, Colombia; 9 Centre of Microbial and Plant Genetics, Department of Microbial and Molecular Systems, KU Leuven, Leuven, Belgium; 10 Laboratory of Gene Technology, Department of Biosystems, KU Leuven, Leuven, Belgium; 11 Institute of Biodiversity, Faculty of Biological Sciences, Cluster of Excellence Balance of the Microverse, Friedrich Schiller University Jena, Jena, Germany; 12 Department of Microbiology, University of Alabama at Birmingham, Birmingham, AL, USA; 13 Department of Microbiology, The Ohio State University, Columbus, OH, USA; 14 Department of Civil, Environmental and Geodetic Engineering, The Ohio State University, Columbus, OH, USA; 15 Center of Microbiome Science, The Ohio State University, Columbus, OH, USA; 16 College of Science and Engineering, Flinders University, Bedford Park, South Australia, Australia; 17 Nuffield Department of Medicine, University of Oxford, South Parks Road, Oxford, UK; 18 Consiglio Nazionale delle Ricerche, Istituto per la Protezione Sostenibile delle Piante, Bari, Italy; 19 Department of Biochemistry, Molecular Biology, Entomology and Plant Pathology, Mississippi State University, Mississippi State, MS, USA; 20 Institut Pasteur, Université Paris Cité, CNRS UMR6047, Archaeal Virology Unit, Paris, France; 21 Theoretical Biology and Bioinformatics, Department of Biology, Science for Life, Utrecht University, CH, Utrecht, the Netherlands

Mining data derived from high-throughput DNA or RNA sequencing approaches, including metagenomics, has led to the discovery of a multitude of uncultivated virus genome sequences^1–12^. These sequences improve our knowledge about the representation of the global virosphere and fuel the expansion and refinement of virus taxonomy. Incorporation of these newly discovered viral sequences into high-quality reference databases adds a bottleneck to virology. For formal taxonomic classification, International Committee on Taxonomy of Viruses (ICTV) guidelines stipulate that genome sequences must be available from a public database. However, the correct use of nomenclature and the inclusion of standardized metadata fields are just as important as the availability of sequence data to enable the use and reuse of the data by the global research community. Here, we present standards and recommendations for the submission of virus genome sequence data to public databases for the purpose of taxonomic classification. These represent a conceptual and practical extension to the Minimum Information about an Uncultivated Virus Genome (MIUViG) standards that include guidelines for reporting the virus origin, genome quality, genome annotation, taxonomic classification, biogeographic distribution and host prediction^13^. Aspects of these standards have been reiterated in a recently published consensus viewpoint statement indicating that viruses inferred from metagenomic sequences require strict quality control before they can be used for taxonomic assignments^14^. The guidelines presented here focus on the MIUViG standards on genome quality and expand on the naming of sequences and their submission to public databases.

The ICTV coordinates the classification of viruses into 15 taxonomic ranks, from species up to realm^15–17^ (Fig. 1). It is important to note that the ICTV is not responsible for the classifications of viruses below the species rank, such as strains, variants, isolates, lineages, genotypes or serotypes within individual species, which are instead generally classified by community consensus over time or by non-ICTV expert groups^18,19^. At the species rank, the ICTV requires that the complete genome sequence of a representative member or “exemplar virus” (isolated or identified by (meta)genomic sequencing) be available as an annotated sequence record in one of the International Nucleotide Sequence Database Collaboration (INSDC) member databases^20^. Practically, this means that the annotated genome sequence of any exemplar virus should be submitted to GenBank (National Center for Biotechnology Information (NCBI)), the European Nucleotide Archive (ENA) or the DNA Data Bank of Japan (DDBJ)^21,22^. This choice was guided by the long-term proven reliability, global accessibility and visibility of INSDC databases. Due to this requirement, at least one fully sequenced virus genome per ICTV-ratified species is now readily available to the global research community and can be used as a reference in comparative genomics analyses.

We note that many complete, coding-complete and incomplete virus genome sequences and genomic fragments are available in public repositories other than INSDC (for example, IMG/VR^12^, BV-BRC^23^, RAST^24^, iVirus^25^ or GISAID^26^), whereas other databases such as the Sequence Read Archive (SRA) and Whole Genome Shotgun (WGS) contain unassembled sequencing reads and unannotated or draft genomes, respectively (example guidance from the NCBI: https://www.ncbi.nlm.nih.gov/sra/docs/submit/ and https://www.ncbi.nlm.nih.gov/genbank/wgs/). Such repositories provide a resource for data mining of virus genome sequences if these genomes are further assembled and annotated^27,28^. By mandating the deposition of annotated sequences into the INSDC databases, the ICTV limits the scattering of exemplar genome sequences across databases and promotes the accessibility of the taxonomically classified exemplar viruses. Furthermore, the close links between the ICTV and INSDC through the NCBI enable better database organization and updating because taxonomy identifiers are persistent and the identifiers are updated routinely with each new ICTV taxonomy release.

A virus genome sequence may be submitted to INSDC databases using the dedicated portals of the NCBI (BankIt or table2asn), ENA (Webin) or DDBJ (Nucleotide Sequence Submission System (NSSS)), either by choosing the submission route for individual complete genomes or through batch submission. If the virus genome sequence was assembled from datasets that were generated by the submitter, submission follows the same protocols as for submission of a virus isolate genome. The sequencing reads should be deposited in the SRA database with the metadata linked through BioProject and BioSample^29^, which contain biological data related to individual initiatives (projects) and descriptions of biological source materials (samples), respectively. Metadata in these databases are provided in structured ontologies, including the Biological Sample Ontology, the Environment Ontology^30^ and the Disease Ontology. Although the availability of raw data cannot be enforced and no mandatory requirements currently exist from the ICTV, submitting such data is a best practice that will be useful for future work, including virus discovery and population genetics studies.

If a genome sequence was assembled from a public dataset, submission to an INSDC database should be done as a Third Party Annotation (TPA), a protocol that was initiated for cases in which the original data do not belong to the submitter (see https://www.insdc.org/submitting-standards/tpa-submission-guidelines/ for details and Tisza and Buck (2021)^7^ for an example). Even when the original dataset is in the public domain, we recommend that—whenever possible—the submitter of a newly (re-)assembled or (re-) annotated genome sequence contact the original data depositor(s) to communicate that the data are being reused.

Practical aspects of submission to INSDC databases, with GenBank as an example, are briefly discussed here and published as a detailed standalone guide in Supplementary Note 1. Practical guidelines for batch submission of Uncultivated Virus Genome (UViG) sequences are provided in Supplementary Note 2.

## Genome completeness and sequence quality

To be considered valid for taxonomic classification, genome sequences should be properly assembled. Assembled genome sequences should be checked for terminal redundancy or other evidence of genome termini^31^, contigs should be checked for chimerism by evaluating the distribution of mapped reads and read pairs, and partially mapped or unmapped reads remaining in the dataset should be assessed and interpreted. The deposited genomes of exemplar viruses should at least be coding-complete, meaning that all open reading frames (ORFs) in the viral genome are fully sequenced^32^, whereas genomic noncoding terminal regions or repeat sequences may be incomplete. Incomplete genome sequences or fragments can still be used to provide context for taxonomic classification, but a coding-complete genome sequence is always required to establish a new taxon. More detailed comments and recommendations on genome sequence completeness can be found in Supplementary Note 1, sections 1 and 3.

### UViG sequence submission and naming

**Fig. 2:**
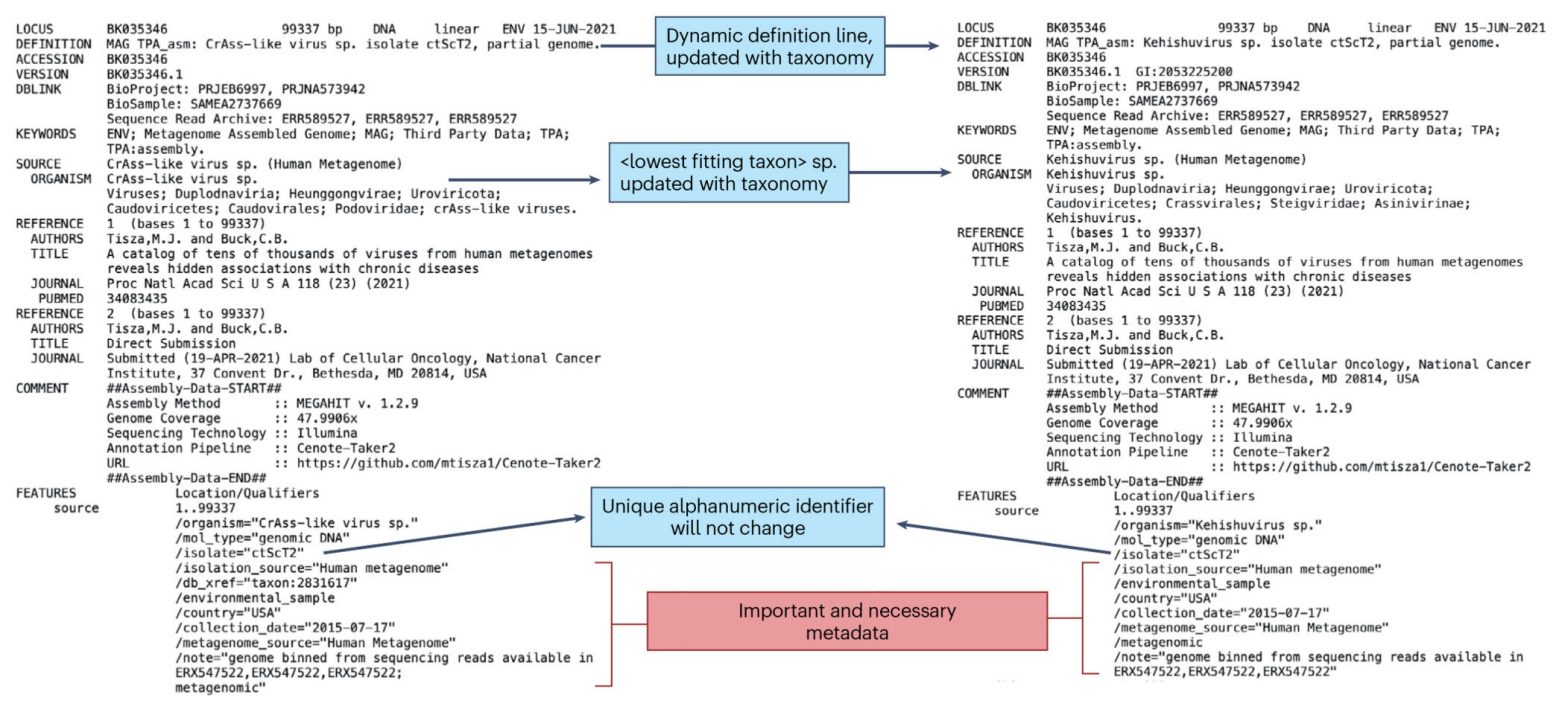
GenBank example of record BK035346. Left: as submitted with the taxonomy at the time of submission; right: updated GenBank record after a later update to the ICTV taxonomy. The ORGANISM name was updated from CrAss-like virus sp. to *Kehishuvirus* sp., now showing the new taxonomic lineage information. The DEFINITION line was updated according to the ORGANISM change.

GenBank requires every sequence record to have a species-rank taxonomic assignment in the <ORGANISM> field. A problem arises when a sequence belongs to a species that was not previously established. In such cases, a species-rank node is created and named according to the format “<lowest fitting taxon> sp.”, in which <lowest fitting taxon> consists of the formal ICTV name of the lowest ranking taxon that can be confidently assigned according to the demarcation criteria and “sp.” for “species” indicates a novel species that has not yet been taxonomically established and named (Fig. 2). Examples are “*Sapovirus* sp.,” “*Herelleviridae* sp.” and “*Cressdnaviricota* sp.”. There is currently no ICTV-approved method to automatically assign a virus query sequence to its lowest fitting taxon because demarcation criteria for assigning sequences to taxa vary widely and should be cross-referenced with taxonomy proposals. Viral ecologists have defined operational clustering of viral sequences into viral operational taxonomic units (vOTUs) based on universal sequence similarity cutoffs^13^, but ICTV-ratified taxa go beyond such preliminary clusters by ensuring some robustness and providing additional information about the members of a taxon. In the GenBank record, metagenomic sequences should be given the /metagenomic, /metagenome_source = “…” and /environmental_sample source qualifiers. If further study shows that some or all the sequences in a metagenomic set have been misclassified, submitters may request an update (https://www.ncbi.nlm.nih.gov/genbank/update/) and GenBank will rename and reclassify the sequences, for example, from “*Siphoviridae* sp.” to “*Vequintavirinae* sp.”. GenBank may also update the organism name in the record, for example, from “*Sapovirus* sp.” to “*Herelleviridae* sp.,” without submitter’s approval if ICTV sequence analysis indicates that a virus containing an “sp.” label has been misfiled.

Using the GenBank record format as a model (Fig. 2), we recommend the following:

- <DEFINITION>: This field is automatically populated from the features in the record using a combination of <ORGANISM> and <ISOLATE> name.
- <ORGANISM>: For UViGs, enter the “<lowest fitting taxon> sp.”. For an isolate, enter the virus name.
- <ISOLATE>: Enter a unique name/code to describe this specific virus genome sequence. Ensure that this field is unique and is unlikely to be used in another study. Do not use taxonomy information in this field, because virus taxonomy is dynamic. As viruses are reclassified, taxonomy information in the <ORGANISM> field will automatically update, but isolate and genome designations are stable over time and hence should not be at odds with taxonomic names. For example, a novel virus <ISOLATE> should not be called “novel flavivirus 5,” as it may turn out not to be a flavivirus in the current or future classification.
- Most databases can, at present, only accommodate the 26 letters of the Medieval Latin alphabet (that is, ISO basic), ten numbers and a few special characters, such as hyphens, underscores and forward slashes. If an official virus name contains Greek letters, special characters or diacritics (for example, Đakrông virus), they can be entered, but be aware that most databases will convert them to the standard Latin-script letters (for example, Dakrong virus) or may even produce an error. The correct spelling in publications should remain Đakrông virus. Underscores and hyphens may be used; forward slashes are typically included in identifiers (IDs) for virus pathogens with formatting requirements, such as members of *Filoviridae*^19^ and *Caliciviridae* and influenza A/B/C/D viruses.
- Critical UViG metadata, including assembly methods and sequence quality descriptors, can be added as structured comments based on the Minimum Information about any (x) Sequence (MIxS) and MIUViG checklists. The most important MIUViG fields are listed in Table 1.
- Do not use a ”complete genome” tag for the virus isolate or genome name unless it has been experimentally verified as complete (including termini determination by, for instance, rapid amplification of complementary DNA ends (RACE)). Currently, the only alternative to”complete genome” in GenBank is “partial genome,” which should be used in the case of UViGs. To specify the genome completeness, wesuggest using the categories from the MIUViG checklist as structured comments, with information about the prediction method provided in the genome metadata (Table 1, Supplementary Note 1).

### Providing appropriate metadata

In INSDC databases, general sequence metadata, such as the origin and source of isolation, are stored as source modifiers (see more detailed description in Supplementary Note 1, section 4). Using the principles of findability, accessibility, interoperability and reusability (FAIR)fordatastewardship^33^,allmetadatafields should be provided as structured ontology terms (following, for example, The Environment Ontology^30^; see also Supplementary Note 1). The minimum recommended source modifiers to be used are <ISOLATION SOURCE>, <COLLECTION DATE> and <COUNTRY>, with <SEGMENT> reserved for viruses with segmented genomes. Additional information specific to UViGs should be provided by submitting a MIUViG sequence^13^ metadata checklist^34,35^ for each UViG sequence and connecting the resulting BioSample package to the UViG genome sequence record by linking the BioSample ID to the GenBank submission. The definition, format and expected values for each field in the MIUViG sequence checklist are available on the Genomic Standards Consortium (GSC) website. We refer to the GenBank Nucleotide record OP880254 as an example of how to implement the MIUViG standards.

### Features

Sequence annotations, such as ORFs, introns, encoded proteins and regulatory elements, are stored as features. Feature annotations should be provided for all UViG sequences that are to be used as exemplar genomes to represent new species. At a minimum, the coding sequences should be specified, including functional annotations based on homology searches, phylogenetic analysis and conserved protein domains, which should be labeled “putative” until experimentally validated.

The availability of complete and consistently annotated records is crucial for the use and reuse of virus sequences and advancing the virology research field. We aim to assist and support the virology community in its expanding use of (meta-)genomic data and the associated taxonomic efforts by promoting the use of this set of standards. Although our recommendations are primarily aimed at viruses inferred from metagenome data (UViGs), they are universally applicable to all viruses. Scientists’ capacity to generate sequences still outpaces our ability to classify them, so submitting new virus data according to these outlined guidelines will greatly facilitate their findability, accessibility and reusability as the ICTV strives to build a robust virus taxonomy.

## Supplementary Material

**Supplementary information** The online version contains supplementary material available at https://doi.org/10.1038/s41587-023-01844-2.

Supplementary Notes

## Figures and Tables

**Fig. 1:**
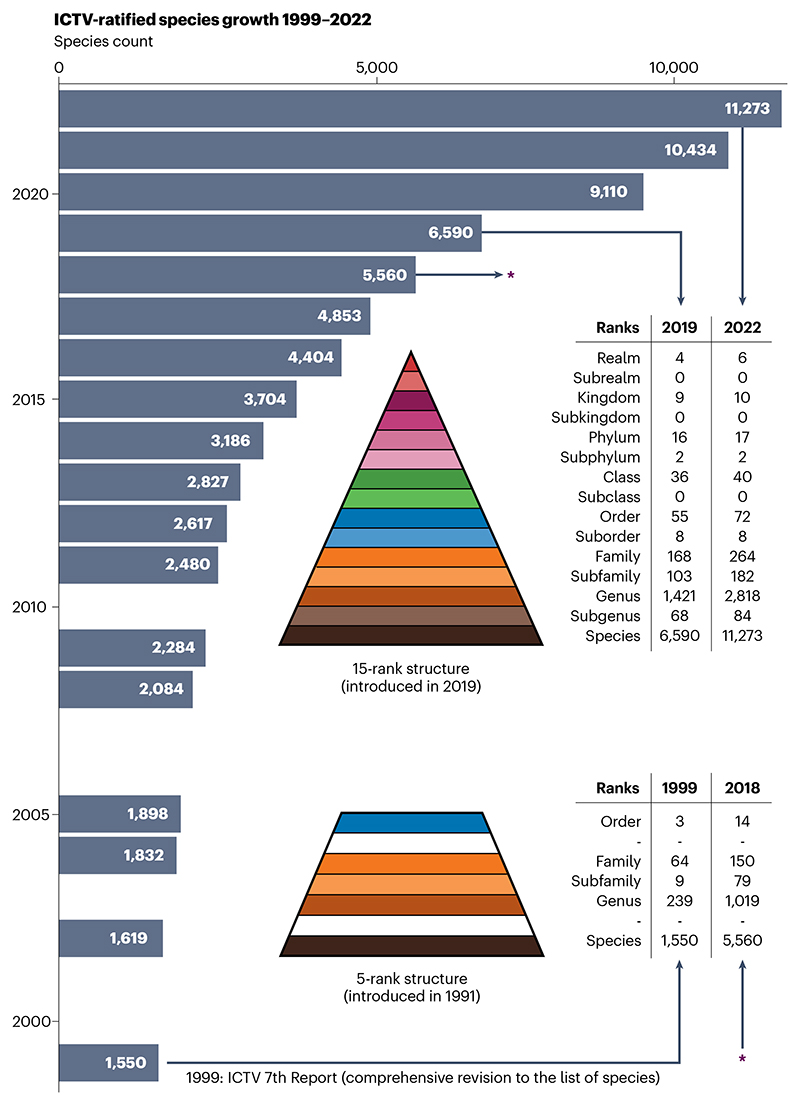
Growth in ICTV-ratified species numbers since the 7th ICTV Report in 1999. The report in 1999 was based on a five-rank structure introduced in 1991. The 15-rank taxonomic structure, which comprises new ranks such as class, phylum, kingdom and realm, was introduced in 2019. This figure illustrates the increase in the number of assigned taxa and the framework for the classification of UViGs.

**Table 1:** Information to provide when submitting UViG sequences to INSDC databases

Information to provide	Where to add	Description	Suggested syntax^a^
Organism	Submission portal + MIUViG checklist structured comment	UViG: lowest-ranking taxon that can be confidently assigned according to ICTV demarcation criteria. Isolated virus: virus name.	[<“lowest fitting taxon” sp.> | virus name]
Isolate	Submission portal + MIUViG checklist structured comment	Unique name or code for this sequence. Do not use taxonomic information here.	<Unique identifier
Source of UViG	MIUViG checklist structured comment	Type of sample used for UViG assembly	[metagenome (not viral targeted) | viral fraction metagenome (virome) | sequence-targeted metagenome | metatranscriptome (not viral targeted) | viral fraction RNA metagenome (RNA virome) | sequence-targeted RNA metagenome | microbial single amplified genome (SAG) | viral single amplified genome (vSAG) | isolate microbial genome | other]
Assembly software	MIUViG checklist structured comment	Tool(s) used for assembly and optionally binning. Include version and parameters.	{software};{version};{parameters}
Assembly quality	MIUViG checklist structured comment	Assembly quality in categories as per the MIUViG criteria. *Finished:* Single, validated, contiguous sequence per replicon without gaps or ambiguities, with extensive manual review and annotation. *High-quality draft genome:* One or multiple fragments, totaling ≥90% of the expected genome or replicon sequence or predicted complete. *Genome fragment(s):* One or multiple fragments, totaling <90% of the expected genome or replicon sequence, or for which no genome length could be estimated.	[Finished genome | High-quality draft genome | Genome fragment(s)]
Completeness score	MIUViG checklist structured comment	(Optional) Estimated completeness of the UViG in percentage.	{quality};{percentage}
Completeness approach	MIUViG checklist structured comment	(Optional) Approach used to estimate completeness, such as identification of terminal repeats or presence of all coding sequences.	{text}
Virus identification software	MIUViG checklist structured comment	Tool(s) used for identification of sequence as virus. Include versions and parameters.	{software};{version};{parameters}
Predicted genome type	MIUViG checklist structured comment	Type of genome predicted for the UViG.	[DNA | dsDNA | ssDNA | RNA | dsRNA | ssRNA | ssRNA (+) | ssRNA (-) | mixed | uncharacterized]

a Entries between []: choose one of the listed descriptors; entries between <>: fill in the UViG or virus information for this record; entries between {}: enter data for your methods used.
