# Supplementary material for: Guidelines for public database submission of uncultivated virus genome sequences for taxonomic classification
Source: Nat Biotechnol. Author manuscript; Available in PMC 2025 Jul 21. (PMC10526704; doi:10.1038/s41587-023-01844-2)
Supplement: Supplementary Notes [file EMS207243-supplement-Supplementary_Notes.pdf]

https://doi.org/10.1038/s41587-023-01844-2

Guidelines for public database submission of uncultivated virus genome sequences for taxonomic classification

In the format provided by the authors and unedited

https://doi.org/10.1038/s41587-023-01844-2

Guidelines for public database submission of uncultivated virus genome sequences for taxonomic classification

In the format provided by the authors and unedited

Supplementary Notes

Supplementary Note 1: Standalone guide for the submission of UViG sequences to the INSDC database

In this guide document, we provide submission examples for GenBank (NCBI)[1]. Submission to the DDBJ and ENA may have slightly different requirements and formats. Please note that data submitted to any of the three resources will be available in all of them, since data is mirrored between the INSDC databases.

1. Genome sequence quality

In 2019, a consensus statement on the Minimum Information criteria for Uncultivated Virus Genome sequences (MIUViG) was published, defining three classes of quality for Uncultivated Virus Genome (UViG) sequences: genome sequence fragments (estimated to be <90% complete), high-quality draft genome sequences (estimated to be ≥90% complete), and complete genome sequences with extensive annotations [2]. The authors of the statement (including several of the authors in this statement) recommended that only complete or coding complete genome sequences can be used as reference (exemplar) genome sequences to establish new species. Genome completeness may be inferred from genomic comparison to related viruses, if the candidate genome can be robustly placed within a cluster of viruses with a well-defined gene content, and/or from the topology of the genome sequence itself, e.g., the detection of direct or inverted terminal repeats. However, estimation of completeness and recovery of complete genome sequences is easier for viruses with circular or circularly permuted genomes than for viruses that have segmented/multipartite genomes, or linear genomes with defined termini. Important to note, virus sequences belonging to all three UviG quality categories may be used to provide additional information for the establishment of new taxa, for example, to test the robustness of phylogenetic trees. Complete or coding-complete genome sequences are necessary, however, to serve as exemplars for the establishment of new species.

2. UViG sequence submission and naming

ICTV is concerned with the naming of virus taxa ranging from species to realms [3], but the naming of individual viruses is outside the ICTV responsibility [4]. Here, we provide a set of recommendations and best practices for the labeling of UViG sequences and submission of metadata.

Submitters should provide unique identifiers (IDs) for each sequence in the <ISOLATE> field, preferably as a single string of at least six alphanumerical characters (e.g., blue53F), using hyphens and underscores to tie separate elements together, e.g., “0815_Eier-kuchen”. Submitters should avoid including common terms like “scaffold” or “contig” in the isolate IDs, or IDs that may be used in other studies (e.g., “soil_virus_contig_01” or “phage_P1”).

Sequences from metagenomic sets should be submitted to GenBank in the <ORGANISM> name format “<lowest fitting taxon> sp.”, in which the <lowest fitting taxon> consists of the formal ICTV taxon name rank (genus or higher) that can be confidently assigned to the sequence, by using the

demarcation criteria for each of these ranks (Figure 1). Examples are "*Sapovirus* sp.", "*Herelleviridae* sp.", and "*Cressdnaviricota* sp." [note that in writing taxon names need to be italicized, but italics are not supported by INSDC databases]. Unique <organism name>s for metagenomic sequences, e.g., "*Sapovirus* sp. Seal/X17", are still acceptable if those <organism name>s have been used in publications, e.g., for viruses of medical importance. GenBank will place these "Taxon sp." Names into unclassified bins reserved for non-ICTV names, e.g., "*Sapovirus* sp." Is found within "unclassified Sapovirus", "*Herelleviridae* sp." In "unclassified *Herelleviridae*", and "*Cressdnaviricota* sp." In "unclassified *Cressdnaviricota*".

In the GenBank record, metagenomic sequences should be given the /metagenomic, /metagenome_source="…" and /environmental_sample source qualifiers. If further study shows that some or all the sequences in a metagenomic set have been misclassified, submitters may request an update (https://www.ncbi.nlm.nih.gov/genbank/update/) and GenBank will rename and reclassify the sequences, e.g., from "*Siphoviridae* sp." To "*Vequintavirinae* sp.". INSDC may also update the organism name in the record, e.g., from "*Sapovirus* sp." To "*Herelleviridae* sp." Without submitter approval if ICTV sequence analysis indicates that a virus containing an "sp." Label has been misfiled.

If a sequence originally submitted with a metagenome name, such as, "*Herelleviridae* sp." Is later used as an ICTV exemplar, the INSDC Taxonomy group at NCBI will rename the <organism name> in the sequence record without requiring submitter approval upon processing the release of the new taxonomy. This information is stored and communicated through the Virus Metadata Resource (VMR, the ICTV file linking the taxonomy with the GenBank accession numbers, https://talk.ictvonline.org/taxonomy/vmr/).

3. Submission recommendations for naming and completeness

In summary, using the GenBank record format as a model (Figure 1), we recommend the following:

- <DEFINITION>: This field is automatically populated from the features in the record using a combination of <ORGANISM> and <ISOLATE> name.
- <ORGANISM>: Enter "<lowest fitting taxon> sp.".
- <ISOLATE>: Enter a unique name/code to describe this specific virus genome sequence. Ensure that this field is unique and is unlikely to be used in another study. Do not use taxonomy information in this field, because virus taxonomy is dynamic. As viruses are reclassified, taxonomy information in the <ORGANISM> field will automatically update, but isolate and genome designations are stable over time and hence should not be at odds with taxonomic names. For example, a novel virus <ISOLATE> should not be called "novel flavivirus 5", as it may turn out not to be a flavivirus in the current or future classification.
- Names should take into account that most databases can, at present, only accommodate the 26 letters of the Medieval (aka ISO basic) Latin alphabet, numbers, and a few special characters, such as, hyphens. If a virus name contains Greek letters, special characters or diacritics (e.g., Đakrông virus), feel free to enter them but be aware that most databases will convert them to the standard Latin-script letters (e.g., Dakrong virus) or produce an error; the correct spelling in publications will remain Đakrông virus. Underscores and hyphens can be used; forward slashes are typically included in IDs for virus pathogens with formatting requirements, such as, members of *Filoviridae* [5], *Caliciviridae*, and influenza viruses.
- Do not use a "complete genome" tag for the virus isolate/genome name unless it has been experimentally verified as complete (including termini determination by, for instance, rapid amplification of complementary DNA [cDNA] ends [RACE]). Genomes that have been

bioinformatically predicted as being complete may be identified as “predicted complete genome”, with information about the prediction method provided in the genome metadata. Note that, in GenBank, the only alternative to “complete” is “partial”, and as a result, the vast majority of UViGs will be tagged as partial genomes. It is the authors’ opinion, that this strict criterion could be reassessed as more computational methods are validated and that the specific MIUViG completeness scores added as structured comments could be used in future to provide more nuance. In GenBank, viral genomes will not be labelled complete if they contain a stretch of 100 or more ambiguous characters.

  - Submit genome metadata via the “Source Modifiers” section of the genome submission process (for general metadata). Additionally, the creation of a separate BioSample for each genome sequence is encouraged using the Minimum Information about any (x) Sequence (MixS) “MIUViG” checklist (for UViG-specific metadata). The metadata fields for UViG quality and completeness (see also Table 1) should be added as structured comments.

4. Providing appropriate metadata

Source modifiers

In INSDC databases, metadata information on a sequence is stored in source modifiers. Using the principles of findability, accessibility, interoperability, and reusability (FAIR) for data stewardship [6], it is best practice to provide as much source metadata as possible, by using structured ontology terms (e.g., The Environment Ontology [7]). Here, we offer guidelines on the implementation of commonly used source modifiers that may be used to provide structured metadata information.

- <HOST> field: Use this field for the host from which the sample was isolated. We recommend not using this source modifier and instead using the MIUViG checklist (see below) for host prediction or the “isolation source” field to provide sample-specific information. If the virus host is predicted from the sequence using computational means [8], the confidence score should be reported (expected precision). Otherwise, leave this field blank. Use the taxonomy IDs from NCBI taxonomy for host description.
- <ISOLATION SOURCE>: Use this field to describe the sample from which the sequence was derived using the Environment Ontology[7] (see also https://www.ebi.ac.uk/ols/index).
- <COLLECTION DATE>: Enter the date of collection for the sample from which the sequence was obtained in the format YYYY-MM-DD.
- <COUNTRY>: Enter the country in which the sample was collected. A standardized list of countries for INSDC submissions can be found here: https://www.ncbi.nlm.nih.gov/genbank/collab/country/.
- <SEGMENT>: For viruses with segmented genomes, this modifier can be used to indicate which segment was recovered. Use this field only if the genomes are similar enough to those of known viruses, i.e., fall within the published demarcation criteria for inclusion into established species for positive segment identification.
- <NOTES>: Note that free text is difficult to computationally parse and is thus not FAIR compliant. Any information that can be entered using structured ontologies as in the fields above is preferred. Use this free text box to add any information that cannot be accounted for in specific source modifiers.

Features

Sequence annotations, such as ORFs, introns, encoded proteins, and regulatory elements, are stored as features in INSDC. Feature annotations should be provided for all UviG sequences that are to be used as exemplar genome sequences to represent new species. At a minimum, the coding

sequences should be provided, including putative functional annotations based on homology searches, phylogenetic analysis, and conserved protein domains. It is good practice to add as many features as can be identified (e.g., transfer RNA [tRNA], terminal repeat regions, promoters).

UViG sequence-specific metadata for BioSample submission

Most often, UViG sequences are accompanied by specific methodological metadata, including the assembly pipeline, viral sequence identification method, completeness estimation, and host prediction. It is critical to attach this information to a UViG genome sequence record, but it does not fit in the standard set of “source” metadata. Moreover, this information is often predicted by bioinformatic programs and thus remains tentative. Instead, this information should be provided by submitting a MIUViG sequence [2] metadata checklist (https://gensc.org/mixs/submit-mixs-metadata/) for each UViG sequence and connecting the resulting BioSample package to the UViG genome sequence record by linking the BioSample ID to the GenBank submission. The definition, format, and expected values for each field in the MIUViG sequence checklist are available on the Genomic Standards Consortium (GSC) website (https://gensc.org/mixs/), with the most important and mandatory ones being:

- <source_uvig>: Type of dataset from which the UviG sequence was obtained, to be selected from “metagenome (not viral targeted)”, “viral fraction metagenome (virome)”, “sequence-targeted metagenome”, “metatranscriptome (not viral targeted)”, “viral fraction RNA metagenome (RNA virome)”, “sequence-targeted RNA metagenome”, “microbial single amplified genome (SAG)”, “viral single amplified genome (vSAG)”, “isolate microbial genome”, and “other”
- <vir_ident_software>: Tool(s) used for the identification of a UviG sequence as a viral genome, such as, the software or protocol name including version number and the used parameters and cutoffs
- <pred_genome_type>: Type of genome predicted for the UviG sequence, to be selected from “DNA”, “dsDNA”, “ssDNA”, “RNA”, “dsRNA”, “ssRNA”, “ssRNA (+)”, “ssRNA (-)”, “mixed”, and “uncharacterized”
- <pred_genome_struc>: Expected structure of the viral genome, to be selected from “segmented”, “non-segmented”, and “undetermined”
- <detec_type>: Type of UviG detected to be selected from “independent sequence (UviG)” (separate contig in dataset), “provirus (UpViG)” (sequenced flanked by host DNA)
- <host_pred_appr> and <host_pred_est_acc>: Tool or approach used for host prediction, and estimated false discovery rates for these tools either computed de novo or from the literature

Supplementary Note 2: Practical guidelines for Batch submission of Uncultivated Virus Genome (UViG) sequences to GenBank

The command-line program `table2asn` allows the quick generation of .asn files for submission to GenBank for thousands of sequences at a time. These .asn files can then be uploaded through BankIt. For submissions of more than 5,000 viruses, submitters are encouraged to contact the database administrators to ensure a smooth submission process.

Template: To run table2asn you will first need to generate a template file (https://submit.ncbi.nlm.nih.gov/genbank/template/submission/). These templates may include the BioProject and BioSample accessions, as well as publication information. This template may then be used when running `table2asn` using `-t` in the command line.

Assembly Information: Assembly information can be incorporated into a structured format that can be added while running `table2asn`. To include the assembly data in the .sqn file, create a tab-delimited table in this format:

```
StructuredCommentPrefix          ## Assembly-Data-START##
Assembly Method                  Unicycler v. 0.4
Genome Coverage                  177x
Sequencing Technology            Illumina; Nanopore
StructuredCommentSuffix          ##Assembly-Data-END##
```

Note that the assembly method script requires "v. " between the algorithm name and its version. If more than one sequencing technology was used, enter both and separate them with a semi-colon.

To include this information when running `table2asn`, use `-w stru_cmt_file` (for which "str_cmt_file" is the name of your assembly information).

Source Information: All source information should be complete and include the mandatory fields isolation_source, collection_date, and country, using the relevant ontologies. Each virus sample must be associated with a unique identifier (as described in this publication) that can be used to separate this virus from other submissions in the database. Source information for viruses inferred from metagenomic data must also contain environmental sample and metagenomic flags and should indicate the type of metagenome from which the samples were obtained (metagenome_source: e.g., fungus metagenome, plant metagenome, gut metagenome).

Source information can be incorporated into the file in multiple ways:

[1] Source information and molecule information can be included in the fasta header of each sequence. For example:
> rainbowtrout_1ct44 [organism=Circovirus sp.] [isolate=ct44] [isolation-source=subsurface seawater] [country=USA] [collection-date=04-Jun-2018] [topology=circular] [BioProject=PRJNAXXXXXX] [BioSample=SAMNXXXXXXXXXX]
Note: If the BioProject and BioSample information were included in the template file, there is no need to include them here.

[2] Some of the source information that is shared by all submissions can be incorporated into the file using the `-j` command in the `table2asn` command line, while source qualifiers unique to each genome can be incorporated using the fasta definition line.
For example:

```
-j [isolation-source=subsurface seawater] [country=USA] [collection-
date=04-Jun-2018]
```

[3] A tab-delimited source modifier table can be created to be read by `table2asn`. The instructions for this table construction can be found at https://www.ncbi.nlm.nih.gov/WebSub/html/help/genbank-source-table.html. Use the file suffix .src and match the prefix to the other files.

Feature Annotation: Genomic feature annotation is recommended, but not required for virus submissions, unless they are being used as International Committee on Taxonomy of Viruses (ICTV) species exemplars. Feature annotation can be incorporated using `table2asn` by including `-f feature.tbl` for which feature.tbl is the name of your table. For this to work, the header in each feature table must match the seqID in the corresponding fasta file; i.e., if the fasta file header is ">abcd1", the corresponding feature table should begin with ">Feature abcd1".

Supplementary Note references

1. Benson DA, Cavanaugh M, Clark K, Karsch-Mizrachi I, Lipman DJ, Ostell J, Sayers EW. GenBank. Nucleic Acids Res 2013; 41:36–42.
2. Roux S, Adriaenssens EM, Dutilh BE, Koonin E V., Kropinski AM, Krupovic M, Kuhn JH, Lavigne R, Brister JR, Varsani A, et al. Minimum Information about an Uncultivated Virus Genome (MIUViG). Nat Biotechnol [Internet] 2019; 37:29–37. Available from: http://www.nature.com/doifinder/10.1038/nbt.4306
3. Gorbalenya AE, Krupovic M, Mushegian A, Kropinski AM, Siddell SG, Varsani A, Adams MJ, Davison AJ, Dutilh BE, Harrach B, et al. The new scope of virus taxonomy: partitioning the virosphere into 15 hierarchical ranks. Nat Microbiol 2020; 5:668–74.
4. Kuhn JH, Jahrling PB. Clarification and guidance on the proper usage of virus and virus species names. Arch Virol 2010; 155:445–53.
5. Kuhn JH, Bao Y, Bavari S, Becker S, Bradfute S, Brister JR, Bukreyev AA, Chandran K, Davey RA, Dolnik O, et al. Virus nomenclature below the species level: A standardized nomenclature for natural variants of viruses assigned to the family Filoviridae. Arch Virol 2013; 158:301–11.
6. Wilkinson MD, Dumontier M, Aalbersberg IjJ, Appleton G, Axton M, Baak A, Blomberg N, Boiten J-W, da Silva Santos LB, Bourne PE, et al. The FAIR Guiding Principles for scientific data management and stewardship. Sci Data [Internet] 2016; 3:160018. Available from: http://www.nature.com/articles/sdata201618
7. Buttigieg PL, Pafilis E, Lewis SE, Schildhauer MP, Walls RL, Mungall CJ. The environment ontology in 2016: bridging domains with increased scope, semantic density, and interoperation. J Biomed Semantics [Internet] 2016; 7:57. Available from: https://jbiomedsem.biomedcentral.com/articles/10.1186/s13326-016-0097-6
8. Edwards RA, McNair K, Faust K, Raes J, Dutilh BE. Computational approaches to predict bacteriophage–host relationships. FEMS Microbiol Rev [Internet] 2016; 40:258–72. Available from: https://academic.oup.com/femsre/article-lookup/doi/10.1093/femsre/fuv048
